# The impact thinking framework: a process for advancing research-to-practice initiatives in neuroaesthetics

**DOI:** 10.3389/fpsyg.2023.1129334

**Published:** 2023-04-25

**Authors:** Susan Magsamen, Tasha L. Golden, Catriona A. Towriss, Joy Allen

**Affiliations:** ^1^The International Arts + Mind Lab, Johns Hopkins University School of Medicine, Baltimore, MD, United States; ^2^Independent Researcher, Cape Town, South Africa; ^3^Berklee College of Music, Boston, MA, United States

**Keywords:** neuroaesthetics, research model, translational research, consensus, framework, interdisciplinary, transdisciplinary, cross-sector

## Abstract

Neuroaesthetics research explores brain, body and behavioral responses to engagement with the arts and other aesthetic sensory experiences. Evidence indicates that such experiences can help address various psychological, neurological and physiological disorders, and that they can support mental and physical well-being and learning in the general population. The interdisciplinary nature of this work contributes to its impact and promise; however, it also creates challenges as various disciplines approach and define research and practice in varied ways. Recent field-wide reports have noted that a consensus translational framework is needed to support further neuroaesthetics research that can deliver meaningful knowledge and interventions. The Impact Thinking Framework (ITF) was designed to meet this need. Through a description of the framework’s nine iterative steps and a presentation of three case studies, this paper posits that the ITF can support researchers and practitioners in understanding and applying aesthetic experiences and the arts to advance health, well-being, and learning.

## Introduction

Neuroaesthetics is an emerging discipline that explores the brain, body, and behavioral responses to engagement with the arts and other aesthetic sensory experiences (Lauring, 2015; Magsamen, 2019). The word aesthetics is derived from the Greek word *aisthetikos*, meaning “sensitive, sentient or pertaining to sense perception,” and it refers to the science of what is sensed through visual, auditory, olfactory, and tactile modalities. *Neuro*aesthetics research, then, is the study of perceptions, emotions, interpretations, and actions that arise while engaging the arts and other sensory experiences.

Driven by recent advances in biomarkers, brain mapping and imaging, neuroaesthetics has become a rapidly expanding research area exploring human experiences associated with music, dance, literature and architecture (Coburn et al., 2017). It is also becoming increasingly interdisciplinary, with relevant research emerging in neuroscience and neurology, as well as in education, psychology, medicine, social science, complementary medicine, pediatrics, gerontology, and more. In short, applied neuroaesthetics research has come to involve a broad range of studies and initiatives at intersections of arts and aesthetics with health, well-being, and learning.

### Growing understanding and impact

Applied neuroaesthetics research ranges from basic scientific discoveries to translational and clinical findings that have relevance from individual to community and policy levels (NeuroArts Blueprint, 2021). For example, studies involving art, music, and dance/movement therapies have found these to be effective in addressing psychiatric issues and neurological disorders, as well as PTSD (Fancourt and Finn, 2019). More broadly, evidence shows that the arts can help reduce mental distress and internalizing symptoms, such as depression and anxiety (Golden et al., 2021), and that aesthetic experiences in healthcare contexts benefit patient healing, patient-healthcare worker relationships, and general experiences of medical and hospital staff (Putrino et al., 2020). The arts have also been found to have positive impacts on a number of neuro-developmental, cognitive, and neurological disorders and can protect against cognitive decline (Balbag et al., 2014; Chancellor et al., 2014).

Studies have also indicated that art and aesthetics can support well-being and learning, with impacts on maternal-infant bonding (Persico et al., 2017), brain development (Fernandez, 2018), language skills (Linnavalli et al., 2018), and early-childhood learning capacity (Golding et al., 2016), as well as improved academic performance and in-school behaviors (Asbury et al., 2008; Yang et al., 2014). Beyond childhood, engagement with the arts and aesthetic experiences has been found to be protective of cognition and mental wellbeing, with positive impacts on self-esteem, self-acceptance, confidence and self-worth, which in turn are protective against stress and mental illness (Franklin, 2013; Grogan et al., 2014; Ascenso et al., 2018).

Given these findings, it is clear that there exists immense potential for research-to-practice applications at individual, interpersonal and society levels (Sonke et al., 2019). Knowledge about neuroaesthetics can support the prevention of ill health, the promotion of learning and well-being, and the management and treatment of disease (Fancourt and Finn, 2019).

### Challenges of a growing field

Unfortunately, while the field’s growing variety of perspectives and backgrounds has the potential to enrich neuroaesthetic inquiry, it can also cause fragmentation. Researchers of different disciplines, as well as arts practitioners who utilize and contribute to research, often lack common ground and a shared language (NeuroArts Blueprint, 2021). They may have varied understandings of existing challenges and how best to tackle them. There is also great heterogeneity in definitions of key terms, outcomes, and research designs and practices between researchers of varied disciplines. For example, the WHO’s global 3000-study review of the arts and health literature found great diversity in study designs in the field, with a paucity of randomized-controlled trials and a lack of innovative designs that can rigorously assess the impact of aesthetic engagements (Fancourt and Finn, 2019; NeuroArts Blueprint, 2021). These factors act as barriers to the synthesis of evidence, which in turn impedes the development of best practices and clinical guidelines, as well as sound evidence-based policy. It additionally hinders the dissemination and scaling-up of successful neuroaesthetic interventions (Golden et al., 2021).

Clearly, a consensus framework is needed to support the highly interdisciplinary nature of research in this field. Indeed, this was affirmed by a 2021 global report titled *NeuroArts Blueprint: Advancing the Science of Arts, Health and Well-Being* (NeuroArts Blueprint, 2021), which aimed to provide a blueprint for developing and accelerating the field of neuroaesthetics (or, in its term, “Neuroarts”). Aiming for an ecosystem in which diverse stakeholders align around common goals at intersections of science, arts, technology, and health, the report recommended strengthening the field by creating models that can bridge research and practice.

In response, this paper introduces the Impact Thinking Framework (ITF) as a model designed to provide a multidisciplinary, rigorous approach to empirical research and translation in neuroaesthetics. Below, the methods used to develop the ITF are described, as well as its main features; this is followed by a presentation of the framework’s nine steps in detail. Finally, the paper offers three case studies that illuminate how the ITF can be applied to neuroaesthetics research and initiatives.

## Framework background

The first iterations of the ITF emerged from an interdisciplinary working group hosted at the International Arts and Mind Lab (IAM Lab) at Johns Hopkins University in 2017. This heterogeneous group consisted of researchers and scholars spanning the sciences, social sciences and humanities; artists of various practices; architects; and experts in design thinking, communication and implementation. The working group was tasked with providing feedback regarding a translational research approach for neuroaesthetics. Through a review of existing research-based models (including implementation science (Nilsen, 2015), action research (Adelman, 1993), theory of change (Reinholz and Andrews, 2020), empirical basic research models (Lewis et al., 2020), science of learning (Weinstein et al., 2018), and design thinking (Razzouk and Shute, 2012; Weinstein et al., 2018), the working group considered the need for a new framework that could support and potentially accelerate neuroaesthetics research and translation.

This process resulted in three key recommendations: First, knowledge generation should be guided by a structured framework that also allows for flexibility and openness. Second, the process should be grounded in interdisciplinary collaboration that enables consideration of all aspects of the issue at hand, both in terms of disciplinary perspective and scale—from the molecular to the societal. Finally, in order to support scaling and sustained implementation of successful interventions, researchers must establish partnerships with practitioners from the outset, and plan to go beyond traditional dissemination efforts. These recommendations were concretized by the working group in the form of the ITF.

## The Impact Thinking Framework (ITF)

The ITF is designed to guide a multidisciplinary team through nine steps of a research-to-practice endeavor.

**FIGURE 1:**
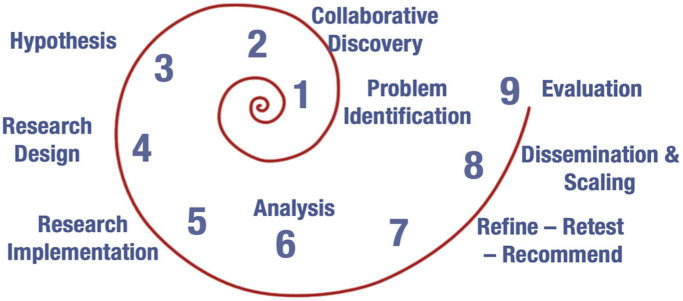
The impact thinking framework.

As shown in the image (Figure 1), Step 1 involves the identification of the problem to be researched, followed by a process of interdisciplinary, collaborative discovery of what is known about the problem, its potential solutions and applications (Step 2). In Step 3, the team selects a hypothesized solution and in Step 4 they design a research study to test that hypothesis. The research is implemented in Step 5, and the data it generates is analyzed in Step 6. Depending on the results of this analysis, the team may undertake a process of refinement and retesting of the solution (Step 7). In Step 8, the results of the research are disseminated to multiple stakeholders and, if the solution was found to be effective, a plan for scaling is created. In Step 9, the process concludes with the design of measures and procedures for ongoing evaluation.

The nine steps of the ITF are presented in a spiral rather than a customary linear fashion, because progress through the framework may not always be sequential. Rather, each step may have implications for “earlier” steps, inviting researchers to circle back in order to render their endeavor more rigorous and effective. In addition, the process as a whole is iterative. Rather than conceiving of a research initiative as having predetermined beginning and end points, the ITF acknowledges that the “end” of a given initiative may well point to a new problem-identification process, collaborative discovery, hypothesis identification, and so on. This iterative approach supports impact by helping researchers think through the implications and directions of an initiative. Finally, the ITF’s spiral shape invites researchers to enter the process at any point. While utilizing the framework from Step 1 through Step 9 may be ideal, doing so is not always possible, nor is it the primary way in which the ITF has been used. Any initiative, at any point in its process, can take up the ITF to support its next steps and to illuminate processes that will bolster effectiveness and impact.

### Features of the ITF

The ITF can be applied by multiple disciplines to a variety of studies in neuroaesthetics, including the development or evaluation of existing initiatives, or the creation and testing of new interventions. It recognizes that arts-based practices are generative, and that responsive research must be expansive while meeting thresholds of quality and rigor. Its emphasis on *impact* and *multidisciplinary engagement* is designed to ensure these two features are considered from the very beginning, so that resources can be put into place to support effective research translation.

#### Prioritizing impact

Perhaps the most notable feature of the ITF is its emphasis on impact—understood as the effective translation of research findings into actionable supports for health, wellbeing, and learning. While basic scientific studies are useful to the field, the ITF supports translational work by building in the expectation of moving research into action and practice. As a result, evaluation, scaling, and multi-audience dissemination opportunities are recognized as core aspects of the process—rather than after-the-fact considerations.

#### Emphasis on multidisciplinarity

Another key feature of the ITF is its emphasis on conducting research with a multidisciplinary team. This approach recognizes the foundational importance—and ongoing challenge—of involving diverse perspectives when conducting research in an inherently multidisciplinary arena such as neuroaesthetics. Multidisciplinary engagement and representation affects every element of the research process, from problem identification (people in different fields will understand and describe a problem in varied ways) through impact (different disciplines may recognize varied practical applications of the research) and dissemination (different fields may have varied means of communicating with researchers, health and arts practitioners, and publics).

To pull together this diversity of perspectives, the ITF offers clear steps that multidisciplinary groups can agree to take together, from problem identification through evaluation and scaling. Such groups will benefit from the ITF’s explicit acknowledgment that, to be successful, multidisciplinary groups must be intentional about reaching consensus regarding problem descriptions, terms, research approaches, and overall aims. In short, the ITF is designed to counteract siloed research, and to cultivate knowledge that benefits from the complex and varied understandings that inevitably emerge from varied training, education, experience, and practice.

#### Researcher-practitioner collaborations

Given its emphasis on bridging research and practice, the simplest collaboration utilizing the ITF may be a partnership between a brain scientist or health researcher, and a practitioner in an arts-based discipline or program. Of course, some initiatives will find value in involving multiple researchers and practitioners, who then engage in the full Impact Thinking process as a group. Beyond core collaborators, users of the ITF may also consider adding advisors and/or stakeholder groups, in order to further supplement knowledge sets, inform research design and analysis, support relevant and effective dissemination, and assist in developing recommendations and best practices. More generally, the steps of the ITF may point to areas in which additional knowledge, skills, or networks are likely to prove valuable. For example, a researcher who lacks dissemination skills beyond academia need not become a communications expert before utilizing the ITF. However, the emphasis that the ITF places on effective dissemination may spur them to engage additional expertise to ensure dissemination goes beyond traditional outlets to reach varied potential “end users” of the initiative’s findings.

### The steps of the ITF

The nine proposed steps of the Impact Thinking Framework are described in detail below. As noted, the steps exist on a spiral, and need not be completed in order. Many entry points are possible, and each step can inform and support the others.

1. **Problem Identification:** This step begins with a meeting that includes both researchers (ideally from more than one discipline) and practitioners who work with the topic in question and can co-identify and co-describe the problem to be investigated. The interdisciplinary nature of this meeting enables attendees to consider a problem from the lens of multiple perspectives and to guide their thinking toward questions that may have greater impact or broader implications. While discussions at this stage may be broad, it is assumed that any identified problems are documented by evidence and that an arts-based process is a feasible solution. By the conclusion of this step, a core team has been formed to work on the initiative (the Impact Team), and the broader problem has been narrowed to a particular set of research questions or problems to solve. While this step’s meetings can be conducted in many ways, a common helpful component is an initial review of data related to the scope and characteristics of the general problem, such as related research and case studies.
2. **Collaborative discovery:** This step is a formalized process designed to generate insights into what is known about the problem, its potential solutions, and their applications from a range of disciplinary perspectives. This critical step is often where interdisciplinarity shines; for example, a given problem may be recognized and addressed by multiple disciplines, but using different terms and processes, with different implications. Thus during Collaborative Discovery, the Team searches broadly for what is known about a given situation, how it is understood and approached by various fields, and who is affected by the issue and its potential solutions. Often combined with Step 1, this step also involves considering what further information the Impact Team needs to access, and whether additional members may need to be invited. This step lays the groundwork for hypothesis development.
3. **Hypothesis:** In the third step, the Impact Team develops and tests the face validity of a number of hypothesized solutions, considering their potential impacts at multiple levels of the social ecological model (Centers for Disease Control and Prevention, 2022). The selected hypothesized solution will ideally include a measurable change and indicators that can be used to gauge impact.
4. **Research design:** In the fourth step, the Impact Team develops a proposed research process to test the selected hypothesis. The Team considers methodologies used by multiple disciplines, as well as in previous neuroaesthetics research. The ITF recognizes that research designs, including measurement tools and assessments, will vary based on the disciplines engaged, the proposed solution(s), and end users of the findings produced.
5. **Research implementation:** This step indicates the time during which research is carried out and documented.
6. **Analysis:** Once data collection is complete, the Impact Team conducts an initial analysis of data, engaging multiple disciplines in the process–as varying backgrounds and experiences are likely to ask varied questions of the data, and notice different patterns.
7. **Refine, retest, recommend:** If initial analysis warrants, Step 7 involves refining and retesting the solution to increase understanding or impact. Once any retesting and subsequent analyses are complete, the Impact Team writes a full report, drawing from documentation made in earlier steps to detail the process. To optimize impact, the report should include recommendations for both practitioners and researchers, as well as for policymakers (when applicable). If an intervention is found to improve health outcomes, the report should also detail the features and conditions of the intervention in such a way as to support implementation by practitioners.
8. **Dissemination and scaling:** As the ITF is a research-to-practice approach, its penultimate step is a multifaceted dissemination effort. In Step 2, the Impact Team identified a broad group of stakeholders and applications for the study. Now, in Step 8, the Team designs and implements an appropriate dissemination plan to these various stakeholders. While academic publishing may be part of the dissemination plan, efforts must also consider practitioner and policy-maker platforms, as well as various media formats.
If a studied intervention is found to be effective, the Impact Team will also use Step 8 to assess opportunities and, if appropriate, develop a plan for scaling the solution. Such a plan may include associative strategies such as training and capacity building; multiplicative strategies such as replication through a proscribed approach; and expansion strategies which include serving more people in the same way.
9. **Evaluation:** If an intervention is sustained or scaled, the Impact Team will evaluate effectiveness over time, identifying responsive measures and processes that capture impact and inform development.

It is worth noting again that, while the steps of the ITF are presented sequentially, Impact Teams can initiate use of the framework at any stage. In addition, while the ITF was designed primarily for research use, it can be applied to projects that do not include traditional research components.

## ITF case studies

While the ITF is new, it has been applied over the last three years to a number of studies and interventions which provide insights into its value for the field of neuroaesthetics. This final section describes three case studies.

### One Book Baltimore

The ITF was applied to an initiative called “One Book Baltimore” (OBB)—which was launched in 2018 as a five-year collaboration between Baltimore City Public Schools (BCPS), the Enoch Pratt Free Library (EPFL), the T. Rowe Price Foundation, and the IAM Lab. The aim was to determine the potential for a literature-based initiative to support the well-being of middle school students throughout the city of Baltimore.

As noted by the ITF, initial meetings among collaborators began with Problem Identification and Collaborative Discovery, which were bolstered by the Impact Team’s varied collaborators (researchers, funders, educators, administrators, youth librarians) and their unique perspectives on middle school students, literature, education, and bibliotherapy initiatives. As these varied perspectives were shared and synthesized, the Team identified and agreed upon a “problem” (the need for middle school students to feel more understood by themselves and others, and more connected with their community); they also generated hypotheses about how a community-wide reading experience may provide some of this understanding and connection.

The spiral nature of the ITF is evident in the story of OBB’s progress. Because it is a multi-year initiative, the first year moved through Design and Implementation, which primarily involved selecting a young adult (YA) novel to be read during the citywide program, disseminating that book throughout dozens of middle schools in the city and hosting public, book-related community events at local libraries. Following the first year’s Analysis stage, the Team refined the Problem Identification process by addressing well-being outcomes that reflected the lived experiences of Baltimore students and generating more targeted Hypotheses.

Analysis in the program’s second year indicated that 85% of student respondents who received OBB’s selected book (*Dear Martin*) (Stone, 2017) read it, 76% said it influenced how they thought about violence and racism, and 65% had discussed the book with friends. In addition, 70% of parent respondents reported discussing the book with their children. In short, the program showed early signs of effectiveness. In addition to producing more recommendations and refinements for the subsequent year, the Impact Team moved into Dissemination and Scaling (Step 8)—with stories and interviews about the program featured on local news outlets such as Baltimore Magazine (Harold, 2018), 3BL Media (2018), and WMAR-2 (2018).

Following the spiral nature of the ITF, experiences of the second year of the program informed another iteration of OBB, with the selection of another book [*Long Way Down* (Reynolds, 2018)] and further refinement of the Hypotheses (Step 3) and Research Design (Step 4). Following the third year of Implementation (Step 5), the Analysis and Refine/Recommend steps revealed the extent to which students had been exposed to community violence (nearly 50% of respondents). It was also revealed that students appreciated the book, found it influential, discussed it with friends, and wanted even more opportunities to engage with the content. Findings and recommendations that will bolster OBB and support similar community-wide reading programs are detailed in a peer-reviewed journal article in Journal of Community Psychology (Golden et al., 2022) and in a comprehensive report delivered to stakeholders in BCPS, EFPL, and the T. Rowe Price Foundation.

Finally, with an eye toward Step 8’s “Dissemination and Scaling,” the Impact Team worked to extend OBB learnings to other communities in the U.S. Researchers have presented findings at multiple academic conferences; librarians have presented at professional convenings; funders and librarians have met with entities pursuing similar initiatives in other regions; and additional news programs have raised public awareness and interest (CBS Baltimore, 2019; WBAL–TV11, 2019).

As a neuroaesthetics initiative examining impacts of literature and literary events on youth well-being, OBB has demonstrated effectiveness in Baltimore, as well as the potential for uptake and development in more regions. The ITF enriched OBB’s development, evaluation, and ongoing refinement by emphasizing multiple disciplines and fields; planning for iteration to increase effectiveness over time; and orienting design and implementation toward impact and scaling. Indeed, its focus on impact has allowed OBB to continually increase engagement, raise awareness and interest across the country, and potentially improve well-being opportunities for middle school students both in Baltimore and beyond.

### Guitar PD

The ITF was applied to “Guitar PD,” a partnership with the Johns Hopkins Center for Music and Medicine. This project brought together neurologists and musicians for a unique series of guitar lessons specifically designed for people with Parkinson’s disease (PD). During an 18-week period, participants with PD were randomly assigned to treatment and control groups and assessed at the outset and every 6 weeks on a variety of self-reported and performance-based measures, including mood, social participation, cognition and arm and hand function. Results showed that the group guitar lessons produced clinically and statistically significant improvement in participant mood and anxiety (Bastepe-Gray et al., 2022).

Following the ITF, Guitar PD began with the establishment of an Impact Team including music therapists, researchers, neurologists and statisticians, along with expert communications and dissemination advisors. Throughout the study, this diverse group explored new ways to communicate with the patient population and, importantly, with their families. New knowledge was gained by these interactions, including identifying a “halo” effect of the intervention on the participants’ family and caregivers–who reported reductions in stress during participation in the study.

In keeping with the goals of the ITF, Guitar PD sought impact via multiple methods of dissemination. For example, study results were shared via a peer-reviewed paper (Bastepe-Gray et al., 2022) and invited scientific talk. The Impact Team also developed a series of ideas to share this work with PD advocacy groups and clinicians, including suggesting specific ways to include guitar lessons in treatment plans. In addition, the Peabody Institute and the Center for Music and Medicine at Johns Hopkins University drew upon this study to develop an ongoing guitar program for people with PD (Peabody Institute, 2022) that is currently thriving and is being replicated in other communities.

The dedicated construction of an interdisciplinary team, supported by the ITF, brought important communications and scaling expertise to the Guitar PD project, and challenged the team to identify ways to share new knowledge with a range of stakeholders. In particular, the ability to initiate new, sustainable programs (such as ongoing guitar lessons for PD patients) required the development of new relationships and community connections, which the ITF helped the Impact Team to consider and develop. Early successes are informing additional evidence-based interventions for the local community, including an ongoing drumming and choral program–the impacts of which are also being measured.

### SmokeLess Break Beats

This initiative was housed within The Berklee Music and Health Innovation Studio (BMHIS), an undergraduate lab in which students are guided to develop music-informed, solution-focused resources for a wide range of healthcare challenges. Titled “SmokeLess Break Beats (SmokeLess Break Personalized Playlists | Nicorette x Spotify, 2022)”, this project was a partnership between Spotify and Nicorette in which students were challenged to develop customizable music-based experiences—“music breaks”—that could help address smokers’ needs and minimize their reliance on cigarettes during the smoking cessation journey.

Following steps one through four of the ITF, the project began by establishing an Impact Team composed of a music therapist with expertise in behavioral health and program development, an addictions specialist, branding/marketing experts, and data scientists. The Impact Team identified nicotine addiction as the problem to be addressed, and used the multi-disciplinary expertise of its members to collaboratively explore the issue and solutions. Having noted the potential for music to support smoking cessation, the Impact Team proposed to create short music-based experiences to promote relaxation, confidence, focus, and physical release to aid the cessation process.

Following this work, students in the BMHIS were added to the Impact Team, and a second iteration of steps one through four began. During this time, the Impact Team collaboratively explored listening preferences and types of music that could facilitate specific emotional and physical states to support a user’s smoking cessation. Students ultimately designed music break interventions (Step 4) by creating playlists with targeted variations in rhythm, lyrical content, and melody designed to generate the user’s desired mood state (relaxation, physical release, confidence, or focus).

In line with the ITF’s focus on scale and impact (Step 8), the project’s partnership with Spotify (a market leader in music sharing) enabled the “music break” experience to reach a mass audience. Spotify has a reliable algorithm, allowing users of the intervention to access a personalized therapeutic playlist using a one-question quiz about their emotional need state. At the time of writing, “SmokeLess Break Beats” has moved into Step 5 of the ITF: implementing a pilot study to determine the effectiveness of the intervention.

In sum, using the ITF within the BMHIS lab provided students with a structure for applying health-related problem-solving to human-centered product design. Its use supported students in gaining foundational knowledge regarding the research base, as well as practical skills in translating knowledge into real-world solutions with measurable impact.

## Conclusion

The emergence of the ITF coincides with increased interest in neuroaesthetics and “Neuroarts,” including recent prominent calls for tools that can support the field’s translational research capacity (NeuroArts Blueprint, 2021). The ITF’s response to those calls, as well as its use in recent initiatives, indicate its alignment with fieldwide discourses and its potential for further implementation. Additional documented use of the ITF is needed to robustly illuminate its applications and value to neuroaesthetics inquiries and initiatives.

This article has presented the Impact Thinking Framework as a tool that can support the field of neuroaesthetics by addressing issues of fragmentation and supporting the multidisciplinary development of scientifically rigorous translational research. Through a description of key features, nine iterative steps, and case studies, this paper has argued that the ITF provides actionable support for the development of impactful neuroaesthetic solutions.

## Data availability statement

The original contributions presented in this study are included in the article/supplementary material, further inquiries can be directed to the corresponding author.

## Author contributions

All authors listed have made a substantial, direct, and intellectual contribution to the work, and approved it for publication.
